# Knowledge, Attitude, and Practice of Dental Practitioners Towards Anterior Teeth Shade Matching in Saudi Arabia

**DOI:** 10.7759/cureus.30505

**Published:** 2022-10-20

**Authors:** Mahesh Suganna, Rayan Fadel Alsharifi, Lara El Jammal, Mennatullah Ahmed Ibrahim, Haya Saif, Merna Mohammed Al Turaiki, Ruba Ahmead Saleh Abu Alhous

**Affiliations:** 1 Department of Prosthodontics, Riyadh Elm University, Riyadh, SAU; 2 College of Dentistry, Riyadh Elm University, Riyadh, SAU

**Keywords:** saudi arabia, attitude, knowledge, dental practitioner, anterior shade matching

## Abstract

Background: When taking into account several factors, including the kind and intensity of the light source, the season, the angle of incidence, the patient's attire, age, gender, and the time of day and year, color is one of the most crucial variables of aesthetic dentistry. The most challenging part of prosthetic dentistry is choosing a replacement tooth's color so that it blends in with the surrounding teeth and covers gingival tissue. As a result of the fact that having an understanding of the concept of color is extremely important in order to achieve good aesthetics, and the distribution of color is highly important to achieve shade matching.

Aim of the study: The goal of this study was to evaluate dental professionals' self-reported knowledge, attitudes, and practices regarding anterior tooth shade matching in Saudi Arabia.

Methodology: This research study was conducted in Saudi Arabia from February to April 2022. Two hundred fifteen registered dental practitioners participated in the cross-sectional, closed-ended questionnaire-based survey. Dental health professionals working in dental colleges, dental clinics, or other dentally significant institutions like hospitals (both government and private) and their respective private practices in various capacities as professors, teachers, or post-graduate students answered the research questionnaire. Dentists who were not involved actively in shade selection or declined to participate in contact through the mail were excluded. Chi-square and Fisher’s exact tests were used for the comparison of categorical data, and a p-value of 0.05 at 95% CI was statistically significant.

Results: Among the questions that were asked, the majority of them said age, sex, gender play, and appointment timing, along with patient opinion play a very important role in shade selection. Moreover, among the patients, 34% noted the manual method of shade selection, and 45.6% of them chose a combination of manual and mechanical methods. When they were asked about the commonest reason they came across for doing a shade selection of anterior teeth, around 52.60% said aesthetics, while 20.90% said fracture of anterior teeth. While 19.1% of people used rubber dam isolation, 34.0% of them preferred the use of cotton rolls and absorbing points, and only 16.7% of them often used Teflon tape. The majority of them had difficulty with shade selection due to the light source, as viewing shades in poor quality light influences how color is perceived; thus, the quality of light is the most influential factor in shade-taking practices. Around 44.7% of them preferred to do shade selection under natural daylight.

Conclusion: Based on the results obtained from the current study, the authors would like to conclude that more attention should be focused on improving the knowledge of color science and its application in aesthetic dentistry, as the color and appearance of teeth are complex phenomena with many factors. Hence, for a good aesthetic outcome to be achieved, the dentist should consider all possible entities that influence shade selection.

## Introduction

Building up a characteristic dental appearance presently constitutes an imperative assignment within the specialties of prosthodontics and traditionalist dentistry [1]. The increase in the aesthetic demands of the patient signifies the importance of color selection in aesthetic dentistry. Many rebuilding efforts fall flat due to a lack of color selection. The choice of the color of lost teeth that harmonizes with the adjoining teeth and encompasses gingival tissue (emergence profile) is the most complex step in prosthetic dentistry [2]. The four essential determinants of an ideal aesthetic treatment result are position, contour, texture, and color of the restoration [1]. Generally, shade is a combination of three variables: hue (H), value (V), and chroma (C). Hue is what recognizes one color from another; value shows the softness of a color extending from unadulterated dark to immaculate white, whereas chroma is the degree of color immersion that portrays the strength, intensity, or vividness [1,2].

Essentially, the method of shade coordinating is carried out utilizing visual and/or instrument strategies [1]. The visual strategy of shade determination is commonly utilized within clinics and training institutions. When using the visual strategy, the tooth and its color should be visible at the same time under the same lighting conditions. Factors such as external light conditions, experience, age, color visual impairment, and weakness of the human eye ordinarily subject visual shade choice strategy to irregularities and bias. Other components that will impact clinical judgment incorporate the make-up worn by patients, the color of the patient's clothes, and the surrounding [1,3].

The instrumental shade coordinating strategy, on the other hand, is objective, quickly obtained, effortlessly evaluated, and can be replicated. These solid and alluring highlights kill the mistakes related to the visual method. The most critical drawback of an instrumental strategy is the cost of the instrument, as each dental practitioner cannot afford the cost of the equipment, especially in developing countries [2]. Currently, there are several available technology-based shade-matching devices. These are colorimeters, spectrophotometers, digital color analyzers/digital cameras, and instruments that combine these technologies. Even though the many instruments approaches for dental shade matching are objective, the usage of these methods is restricted due to the high cost of the equipment and the challenges involved in its operation. Therefore, both students and professionals need to have an understanding of the responsibilities they play in achieving the aesthetic needs and expectations of patients, particularly when it comes to the placement of anterior restorations [1].

It is therefore important that, as part of the future workforce, this knowledge, attitude, and practice (KAP) survey will provide baseline data and identify gaps that may facilitate understanding and further action to plan, implement, and evaluate practice toward anterior teeth shade matching among dental practitioners in Saudi Arabia. Hence, the purpose of this study is to assess the knowledge, attitudes, and practices of dental practitioners in Saudi Arabia with regard to anterior tooth shade matching, as stated by themselves, and by means of this investigation, establish the importance of the same.

## Materials and methods

This research study was conducted in Saudi Arabia from February to April 2022. As per our initial hypotheses, we aimed to evaluate dental professionals' knowledge, attitudes, and practices regarding anterior tooth shade matching in Saudi Arabia, as expressed by the professionals themselves, and use this investigation to establish the significance of the practice. Two hundred and fifteen registered dental practitioners participated in the cross-sectional close-ended questionnaire-based survey. This sample size was agreed upon after using the Fisher formula for sample size calculation, with the formula being: sample size= Z^2^P (1-P)/ D^2^, where Z = coefficient of Z statistics obtained from a standard normal distribution, P = Proportion (in %) Q = 1 − P, and D = sample error tolerated (in %).

The research was distributed among Dental Health specialists either working in dental colleges, dental clinics, or through other institutions of dental significance such as hospitals (both government and private) and their respective private practices in various capacities as professors, teachers, or post-graduate students. The inclusion criteria for our investigation were that we included only those dental students and professionals who were willing to participate, and in terms of the exclusion criterion, those who were not willing to participate or had submitted the questionnaire with incomplete data were rejected from the domains of the study.

The study participants were informed about the purpose and objective of the research, and informed consent was obtained. The ethical clearance was obtained with application number FIRP/2021/112 and IRB approval number: FIRP/2021/112/627/605. Data were entered and analyzed using IBM Corp. Released 2017. IBM SPSS Statistics for Windows, Version 25.0. Armonk, NY: IBM Corp. A descriptive analysis of data was followed by inferential statistics. Chi-square and Fisher’s exact tests were used for the comparison of categorical data. A p-value of ≤ 0.05 at 95% CI was considered statistically significant.

## Results

Table 1 shows the description of 215 study participants of dentists in Saudi Arabia. 47% (n=101) of dentists were males and 53% (n=114) were females.

**Table 1:** Characteristics of the study participants (N=215)

Variables		n	%
Age	20-29	96	44.7
	30-39	78	36.3
	≥40	41	19.1
Gender	Male	101	47.0
	Female	114	53.0
Address	Central	103	47.9
	East	31	14.4
	West	32	14.9
	South	31	14.4
	North	18	8.4
Qualification	Bds	100	46.5
	Masters	84	39.1
	Ph.D.	31	14.4
Main Work area	Faculty Dental college	68	31.6
	Private practice	88	40.9
	Govt, Job	59	27.4
Experience (Years)	0-5	92	42.8
	6-10	54	25.1
	>10	69	32.1
Specialization in Maxillofacial Prosthodontics & Implantology	Yes	64	29.8
	No	151	70.2

The percentage of participants between the age of 20-29 years old is 44.7%, 30-39 years old is 36.3%, and≥40years old is 19.1%. The participants were respectively 8.4%, 14.4%, 14.4 %, 14.9 and 47.9% from North, South, East, West, and central Riyadh geographical zones. Among the 215 dentists 46.5 % (n=100) were BDS, 39.1 % (n=84) were MDS and 14.4%(n=31) were PhD qualified. Out of the 215 dentists, 13.6% were working as faculty in dental colleges, 40.9% as private practitioners, and 27.4% as government practitioners. We had 29.8% specialized in maxillofacial prosthodontists and implantology. The total length of dental practice is between 0-5 years for 42.8% of dentists, 6-10 years for 25.1 % of dentists, and >10 years of practice for 32.1% of dentists.

Table 2 shows the responses to a questionnaire for the knowledge of teeth shade matching and the percentage distributions of the responses.

**Table 2:** Anterior teeth shade matching knowledge (N=215)

Item	Questions	Responses	n	%
1	Essential during shade selection	Knowledge	71	33.00
		Talent	32	14.90
		Skill	59	27.40
		Individual observation	53	24.70
2	Major role in shade selection	Hue	61	28.40
		Value	55	25.60
		Chroma	56	26.00
		Translucency	43	20.00
3	Most affects the color perception	Light source	83	38.60
		Tooth, textures and layers	79	36.70
		Environment	34	15.80
		Receiver (eye)	19	8.80
4	The ideal amount of light required during shade selection	1000 lux	41	19.10
		1500 lux	110	51.20
		2000 lux	52	24.20
		2500 lux	12	5.60
5	Ideal time required for shade selection	<5 seconds	64	29.80
		5-10 seconds	80	37.20
		10-15 seconds	49	22.80
		15-20 seconds	22	10.20
6	Shade selection at the beginning of an appointment is better than doing it later	Yes	184	85.60
		No	31	14.40
7	Age, sex, and gender of the patient play an important role in the shade selection	Yes	187	87.00
		No	28	13.00
8	More concerned about aesthetics	Young male	14	6.50
		Young female	131	60.90
		Adult male	22	10.20
		Adult female	48	22.30
9	Most common complaint patients have regarding anterior teeth restoration	Esthetic correction	113	52.60
		Fractured tooth	45	20.90
		Dental caries	22	10.20
		Failed restoration	35	16.30

For the question “what is essential during shade selection?” 33% of dentists answered: “Knowledge,” 14.90% answered: “Talent,” 27.40% answered “Skill,” whereas almost 24.70% answered: “Individual Observation.” For the question “what is the major role in shade selection?”, 28.40 % of dentists answered: “Hue,” 25.60% answered “value,” 26% answered: “chroma,” whereas 20% of dentists answered: “Translucency.” For the question “Which most affects color perception?”, 38.60% of dentists answered: “light source,” 36.70% of dentists answered: “tooth, textures, and layers,” 15.80% of dentists answered: “environment,” and 8.80% of dentists answered: “Receiver (eye).’’ For the question “what is the ideal time required for shade selection,” 29.80% of dentists answered: “more than 5 seconds,” 37.20% of dentists answered: “5 to 10 seconds,” 22.80% of dentist answered: “10 to 15 second” and 10.20% answered: “15 to 20 seconds.” For the question “Is shade selection at the beginning of the appointment better than doing it later,” 85.60% of dentists answered: “yes,” and 14.40% of dentists answered: “no.” For the question “is age, sex, and gender of the patient play an important role in the shade selection?”, 87% answered: “yes” and 13% answered: “no.’’ For the question “who is concerned more about aesthetics?”, 6.50% answered: “young males” 60.90% answered: “young females,’’ 10.20% answered: "adult male’’ and 22.30% answered: “adult female.’’ For the question “what are the most common complaints patients have regarding anterior teeth restoration” 52.60% of dentists answered: “aesthetic correction,” 20.90% answered: “fractured tooth” 10.20% answered: "dental caries’’ and 16.30% answered: “failed restorations.” 

Table 3 shows the responses to each of the attitude and practice items of the shade selection questionnaire and the percentage distributions of the responses.

**Table 3:** Anterior teeth shade matching attitude and practices (N=215)

Items	Questions		n	%
10	Take patient's opinion during shade selection	Yes	181	84.2
		No	34	15.8
11	Preferred shade selection method	Visual (Manual)	73	34.0
		Instrumental (Mechanical)	44	20.5
		Combination of both	98	45.6
12	In the manual method type of light used	Dental chair light	14	6.5
		Fluorescent light	14	6.5
		Natural daylight	96	44.7
		Natural and Dental Light	58	27.0
		Natural Light and fluorescent light	33	15.3
13	Method of isolation during shade selection	Rubber Dam Isolation	41	19.1
		Cotton rolls and Absorbent wafers	73	34.0
		Teflon Tape	36	16.7
		Evacuator system & saliva ejector	65	30.2
14	While matching the shade of a tooth was done as	A Single Unit	36	16.7
		Two halves	50	23.3
		Cervical third, middle third, incised third	129	60.0
15	Most common shade you use in your practice	A1	31	14.4
		A2	90	41.9
		A3	29	13.5
		A4	19	8.8
		Combination shades	46	21.4

For the question, “do you take a patient’s opinion while doing shade selection? ”15.8% of dentists answered: “No”, whereas almost 84.2% answered: “Yes”. For the question “which method do you prefer during shade selection?”, 34% of dentists answered: “visual (manual)”, whereas 20.5% dentists answered: “instrumental (mechanical)” and 45.6% of dentists answered:” a combination of both. For the question “if manual, under what type of light would you do shade selection?”, 6.5% of dentists answered: “dental chair light and fluorescent light”, 44.7% dentists answered: “natural day light”, 27% of dentist answered: “natural and dental light”, and 15.3% of dentists answered: “Natural and fluorescent light”. For the question “what method of isolation do you prefer while doing shade matching for anterior restoration”, 19.1% of dentists answered: “Rubber dam isolation”, 34% of dentists answered: “cotton rolls and absorbent wafers”,16.7% of dentists answered: “Teflon tape “and 30.2% answered: “Evacuator system and saliva ejectors”. For the question “while matching the shade of a tooth, it is done as...?”16.7% of dentists answered: “A single unit”, 23.3% of dentists answered “two halves” and 60% of dentists answered: “cervical third, middle third, incisal third”. For the question “what is the most common shade you use in your practice?”14.4% of dentists answered: “A1”, 41.9% answered: “A2”, 13.5% of dentists answered: “A3”, 8.8% of dentists answered: “A4” and 21.4% of dentists answered “A combination of color”.

An association between the knowledge of tooth shade characteristics with the age, gender, and qualification of dentists showed no significant correlation (p>0.05) (Table 4).

**Table 4:** Association of knowledge tooth shade characteristics of age, gender, and qualification of participants *: statistically significant

Item	Responses	Age			Gender		Qualification		
		20-29	30-39	≥40	Male	Female	BDS	Mast	PH.D
Item 1	Knowledge	35.4	33.3	26.8	34.7	31.6	37.0	31.0	25.8
	Talent	17.7	14.1	9.8	11.9	17.5	11.0	16.7	22.6
	Skill	21.9	32.1	31.7	25.7	28.9	25.0	28.6	32.3
	Individual observation	25.0	20.5	31.7	27.7	21.9	27.0	23.8	19.4
	p value	0.512		0.525			0.593		
Item 2	Hue	35.4	24.4	19.5	30.7	26.3	31.0	29.8	16.1
	Value	21.9	28.2	29.3	28.7	22.8	24.0	25.0	32.3
	Chroma	25.0	26.9	26.8	23.8	28.1	22.0	28.6	32.3
	Translucency	17.7	20.5	24.4	16.8	22.8	23.0	16.7	19.4
	p value	0.553			0.488		0.569		
Item 3	Light source	40.6	39.7	31.7	45.5	32.5	41.0	41.7	22.6
	Tooth, textures and layers	33.3	39.7	39.0	29.7	43.0	35.0	34.5	48.4
	Environment	14.6	15.4	19.5	14.9	16.7	12.0	16.7	25.8
	Receiver (eye)	11.5	5.1	9.8	9.9	7.9	12.0	7.1	3.2
	p value	0.725			0.151		0.164		
Item 4	1000 lux	25.0	12.8	17.1	17.8	20.2	21.0	14.3	25.8
	1500 lux	51.0	51.3	51.2	44.6	57.0	53.0	51.2	45.2
	2000 lux	18.8	28.2	29.3	30.7	18.4	19.0	32.1	19.4
	2500 lux	5.2	7.7	2.4	6.9	4.4	7.0	2.4	9.7
	p value	0.325			0.125		0.193		
Item 5	<5 seconds	30.2	35.9	17.1	32.7	27.2	32.0	27.4	29.0
	5-10 seconds	34.4	33.3	51.2	40.6	34.2	36.0	35.7	45.2
	10-15 seconds	26.0	20.5	19.5	18.8	26.3	24.0	22.6	19.4
	15-20 seconds	9.4	10.3	12.2	7.9	12.3	8.0	14.3	6.5
	p value	0.327			0.328		0.751		
Item 6	Yes	87.5	83.3	85.4	86.1	85.1	87.0	82.1	90.3
	No	12.5	16.7	14.6	13.9	14.9	13.0	17.9	9.7
	p value	0.738			0.827		0.465		
Item 7	Yes	89.6	87.2	80.5	84.2	89.5	87.0	86.9	87.1
	No	10.4	12.8	19.5	15.8	10.5	13.0	13.1	12.9
	p value	0.349			0.248		1.000		
Item 8	Young male	7.3	6.4	4.9	5.9	7.0	6.0	7.1	6.5
	Young female	61.5	57.7	65.9	65.3	57.0	65.0	60.7	48.4
	Adult male	7.3	12.8	12.2	9.9	10.5	5.0	11.9	22.6
	Adult female	24.0	23.1	17.1	18.8	25.4	24.0	20.2	22.6
	p value	0.848			0.620		0.175		
Item 9	Esthetic correction	55.2	53.8	43.9	56.4	49.1	55.0	51.2	48.4
	Fractured tooth	17.7	21.8	26.8	17.8	23.7	14.0	28.6	22.6
	Dental caries	7.3	14.1	9.8	10.9	9.6	9.0	10.7	12.9
	Failed restoration	19.8	10.3	19.5	14.9	17.5	22.0	9.5	16.1
	p value	0.358			0.627		0.139		

The results of our study have shown that the specialty of the dentists would impact the choice of shade selection between knowledge, talent, skill, and individual observation (p<0.05). Moreover, the hue, value, chroma, and translucency differed from each dentist's experience (p<0.05) (Table 5).

**Table 5:** Association of knowledge tooth shade and experience, workplace and specialty of participants *: statistically significant

Variables		Experience			Main workplace			Specialty	
		<5	6-10	>10	Dental Faculty	Private practice	Govt, Job	Yes	No
Item 1	Knowledge	40.2	29.6	26.1	39.7	26.1	35.6	25	36.4
	Talent	14.1	13	17.4	20.6	11.4	13.6	25	10.6
	Skill	23.9	33.3	27.5	20.6	34.1	25.4	28.1	27.2
	Individual observation	21.7	24.1	29	19.1	28.4	25.4	21.9	25.8
	p value	0.538			0.196			0.039^*^	
Item 2	Hue	32.6	37	15.9	29.4	29.5	25.4	20.3	31.8
	Value	19.6	33.3	27.5	19.1	23.9	35.6	29.7	23.8
	Chroma	26.1	18.5	31.9	35.3	22.7	20.3	35.9	21.9
	Translucency	21.7	11.1	24.6	16.2	23.9	18.6	14.1	22.5
	p value	0.035^*^			0.215			0.052	
Item 3	Light source	41.3	44.4	30.4	26.5	38.6	52.5	18.8	47
	Tooth textures and layers	35.9	38.9	36.2	41.2	37.5	30.5	45.3	33.1
	Environment	14.1	11.1	21.7	25	13.6	8.5	29.7	9.9
	Receiver (eye)	8.7	5.6	11.6	7.4	10.2	8.5	6.3	9.9
	p value	0.465			0.046^*^			<0.001^*^	
Item 4	1000 lux	17.4	20.4	20.3	26.5	15.9	15.3	18.8	19.2
	1500 lux	56.5	42.6	50.7	50	53.4	49.2	51.6	51
	2000 lux	22.8	27.8	23.2	19.1	25	28.8	21.9	25.2
	2500 lux	3.3	9.3	5.8	4.4	5.7	6.8	7.8	4.6
	p value	0.660			0.595			0.796	
Item 5	<5 seconds	33.7	31.5	23.2	22.1	31.8	35.6	31.3	29.1
	5-10 seconds	35.9	33.3	42	41.2	35.2	35.6	35.9	37.7
	10-15 seconds	23.9	24.1	20.3	29.4	21.6	16.9	23.4	22.5
	15-20 seconds	6.5	11.1	14.5	7.4	11.4	11.9	9.4	10.6
	p va;lue	0.543			0.465			0.979	
Item 6	Yes	87	88.9	81.2	92.6	80.7	84.7	87.5	84.8
	No	13	11.1	18.8	7.4	19.3	15.3	12.5	15.2
	p value	0.425			0.106			0.602	
Item 7	Yes	88	87	85.5	80.9	85.2	96.6	87.5	86.8
	No	12	13	14.5	19.1	14.8	3.4	12.5	13.2
	pp value	0.894			0.026^*^			0.882	
Item 8	Young male	5.4	5.6	8.7	7.4	4.5	8.5	6.3	6.6
	Young female	65.2	59.3	56.5	58.8	58	67.8	56.3	62.9
	Adult male	3.3	9.3	20.3	7.4	15.9	5.1	17.2	7.3
	Adult female	26.1	25.9	14.5	26.5	21.6	18.6	20.3	23.2
	p value	0.018^*^			0.291			0.186	
Item 9	Esthetic correction	54.3	59.3	44.9	51.5	52.3	54.2	37.5	58.9
	Fractured tooth	18.5	13	30.4	32.4	11.4	22	35.9	14.6
	Dental caries	6.5	13	13	5.9	13.6	10.2	18.8	6.6
	Failed restoration	20.7	14.8	11.6	10.3	22.7	13.6	7.8	19.9
	p value	0.108			0.025^*^			<0.001^*^	

The workplace and specialty of the dentists made a difference while matching the shade of the tooth by choosing the desired light source, tooth texture and layers, environment, and receiver eye. The age and gender of the patients proved to be critical in shade selection according to the experience of dental practitioners (p<0.05), and also the results showed that according to the experience of dentists in shade selection, young female patients showed more consideration of aesthetic correction with (p<0.05). Moreover, the most common complaint between the workplace and the specialty of dentists regarding anterior teeth restoration was an aesthetic correction, with 59% between fractured teeth, dental caries, and failed restorations (p<0.05).

The results for the gender and qualifications of the dentists would impact the choice of shade selection between visual, mechanical, and a combination of both methods (p<0.05). Moreover, the qualification of the dentists had a significant impact on the choice of background light while choosing shade selection (p<0.05). The qualifications of the dentists made a difference while matching the shade of the tooth by dividing the color either as a single unit, two halves, or as cervical third, middle third, or incisal third (Table 6).

**Table 6:** Participant’s attitude and practices towards tooth shade selection based on age, gender and specialty.

Variables		Age			Gender		Qualification		
		20-29	30-39	≥40	Male	Female	BDS	MASTERS	PH.D
Item 10	Yes	83.3	84.6	85.4	82.2	86	82	89.3	77.4
	No	16.7	15.4	14.6	17.8	14	18	10.7	22.6
	p value	0.948			0.448		0.216		
Item 11	Visual (Manual)	41.7	28.2	26.8	46.5	22.8	37	31	32.3
	Instrumental (Mechanical)	17.7	24.4	19.5	11.9	28.1	12	25	35.5
	Combination of both	40.6	47.4	53.7	41.6	49.1	51	44	32.3
	p value	0.270			<0.001^*^		0.038^*^		
Item 12	Dental chair light	6.3	6.4	7.3	6.9	6.1	7	6	6.5
	Fluorescent light	8.3	5.1	4.9	6.9	6.1	11	2.4	3.2
	Natural daylight	45.8	46.2	39	49.5	40.4	51	41.7	32.3
	Natural and Dental Light	30.2	19.2	34.1	18.8	34.2	21	28.6	41.9
	Natural Light and fluorescent light	9.4	23.1	14.6	17.8	13.2	10	21.4	16.1
	p value	0.304			0.160		0.044^*,^		
Item 13	Rubber Dam Isolation	20.8	16.7	19.5	17.8	20.2	24	16.7	9.7
	Cotton rolls and Absorbent wafers	29.2	34.6	43.9	39.6	28.9	32	34.5	38.7
	Teflon Tape	15.6	17.9	17.1	9.9	22.8	14	16.7	25.8
	Evacuator system & saliva ejector	34.4	30.8	19.5	32.7	28.1	30	32.1	25.8
	p value	0.609			0.054		0.474		
Item 14	A Single Unit	20.8	15.4	9.8	18.8	14.9	25	10.7	6.5
	Two halves	21.9	24.4	24.4	20.8	25.4	22	22.6	29
	Cervical third, middle third, incised third	57.3	60.3	65.9	60.4	59.6	53	66.7	64.5
	p value	0.603			0.610		0.042^*^		
Item 15	A1	12.5	15.4	17.1	10.9	17.5	10	17.9	19.4
	A2	49	32.1	43.9	47.5	36.8	50	35.7	32.3
	A3	11.5	20.5	4.9	14.9	12.3	14	13.1	12.9
	A4	8.3	9	9.8	8.9	8.8	7	9.5	12.9
	Combination shades	18.8	23.1	24.4	17.8	24.6	19	23.8	22.6
	p value	0.299			0.342		0.520		

The specialty of the dentists would impact the choice of shade selection between visual, mechanical, and a combination of both methods (p<0.05). Moreover, the specialty of the dentists had a significant impact on the choice of background light while choosing shade selection (p<0.05). The experience and specialty of the dentist had a significant impact depending on the method of isolation used while choosing shade selection p (<0.05). Moreover, the specialty of the dentist would impact the selection of the most shade colored used in dental practice (Table 7).

**Table 7:** Participant’s attitude and practices towards tooth shade selection based on experience, work area and specialty. *: statistically significant

Variables		Experience			Main work area			Specialty	
		1-5	6-10	>10	Dental faculty	Private practice	Govt, Job	Yes	No
Item 10	Yes	83.7	88.9	81.2	85.3	83.0	84.7	82.8	84.8
	No	16.3	11.1	18.8	14.7	17.0	15.3	17.2	15.2
	p value	0.499			0.915			0.719	
Item 11	Visual (Manual)	37.0	38.9	26.1	36.8	29.5	37.3	25.0	37.7
	Instrumental (Mechanical)	18.5	13.0	29.0	19.1	23.9	16.9	39.1	12.6
	Combination of both	44.6	48.1	44.9	44.1	46.6	45.8	35.9	49.7
	p value	0.191			0.769			<0.001^*^	
Item 12	Dental chair light	6.5	7.4	5.8	5.9	6.8	6.8	1.6	8.6
	Fluorescent light	8.7	5.6	4.3	4.4	10.2	3.4	4.7	7.3
	Natural daylight	43.5	55.6	37.7	32.4	52.3	47.5	28.1	51.7
	Natural and Dental Light	31.5	22.2	24.6	39.7	17.0	27.1	39.1	21.9
	Natural Light and fluorescent light	9.8	9.3	27.5	17.6	13.6	15.3	26.6	10.6
	p value	0.065			0.069			<0.001^*^	
Item 13	Rubber Dam Isolation	20.7	18.5	17.4	27.9	17.0	11.9	18.8	19.2
	Cotton rolls and Absorbent wafers	23.9	42.6	40.6	30.9	34.1	37.3	39.1	31.8
	Teflon Tape	15.2	11.1	23.2	17.6	15.9	16.9	25.0	13.2
	Evacuator system & saliva ejector	40.2	27.8	18.8	23.5	33.0	33.9	17.2	35.8
	p value	0.030^*^			0.365			0.023^*^	
Item 14	A Single Unit	20.7	20.4	8.7	17.6	15.9	16.9	14.1	17.9
	Two halves	22.8	25.9	21.7	32.4	18.2	20.3	28.1	21.2
	Cervical third, middle third, incised third	56.5	53.7	69.6	50.0	65.9	62.7	57.8	60.9
	p value	0.217			0.249			0.498	
Item 15	A1	16.3	13.0	13.0	17.6	12.5	13.6	23.4	10.6
	A2	46.7	44.4	33.3	41.2	39.8	45.8	28.1	47.7
	A3	9.8	16.7	15.9	14.7	15.9	8.5	17.2	11.9
	A4	9.8	5.6	10.1	7.4	9.1	10.2	9.4	8.6
	Combination shades	17.4	20.4	27.5	19.1	22.7	22.0	21.9	21.2
	p value	0.573			0.924			0.037^*^	

## Discussion

One of the most challenging branches of dentistry is aesthetic dentistry [4]. Color is one of the most important determinants of aesthetic dentistry, considering a variety of parameters such as the kind and intensity of the light source, the time of the day and year, the angle of incidence, and the patient's clothing, age, and gender [5,6]. The correct evaluation of tooth color is a critical stage in the proper planning and execution of dental procedures [7]. The shade selection is highly subjective due to a lack of standardization [3,8]. Shade matching is a multifaceted phenomenon that includes both subjective and objective factors [7,9]. Shade matching is much more challenging than it appears, especially when it comes to hue, value, and chroma. However, via training, activity, and experience, the capacity to determine color increases over time [7]. It is highly recommended to select the shade at the beginning of the procedure because when the teeth get dry chroma value changes, and this is confirmed by our survey that showed a majority of the clinician select the shade prior to preparation [9-11].

According to Wager et al., who stated that anytime an object is observed for longer than 10 seconds, the color vision capability of the eyes rapidly degrades, and the perceived color does not stay the same; our analysis revealed that clinicians take almost 5 to 10 seconds while choosing the shade [11].

The capacity to choose the correct color shade is thought to be gender dependent, with women having less color vision deficit [3]. The visual technique is a widely used technique that employs the Munsell color system, which is a widely utilized system [12]. The Munsell color order system is shown in three dimensions, which correspond to the three perceptual components of human color vision (hue, value, and chroma). The visual tooth color determination technique is based on the human eye, which can discern very small color differences [8]. Any deficiencies in shade selection accuracy may be addressed by the application of the instrument technique, which ensures accuracy based on objective criteria and scientific research [11]. The gold standard for assessing tooth color and providing high levels of agreement for the use of shade selection in dentistry is the spectrophotometer method of shade evaluation [4]. Dentists should encourage their patients to choose the color of their prosthetics on their own while providing them with enough expert information to help them make an informed decision [13]. In our investigation, the percentage of male respondents was lower (47%), although the response rate among older people was higher. The educational level was the primary focus, and postgraduate dentists accounted for the biggest percentage of participants. Contrary to the results of our survey, where 26% of respondents said "chromas were the highest score," 143 (81.3%) respondents preferred that the hue play no significant part in shade choosing.

When we compared the essential component used to choose the shade, it was found that the skill factor had the highest percentage (41%) [14]. However, in our study, the knowledge factor had the highest percentage (33%). 37.20% of dentists said, "5 to 10 seconds as the largest proportion," when asked when it is best to choose the color. Candidates stated that the best time to choose a shade is in the morning, with 48.6% agreeing and 44.1% stating that they check a shade twice before settling on a final hue. In our analysis, the most common form of isolation was 34%, with cotton rolls receiving the largest proportion of votes. In a study by Alruwaili et al., 45.9% of the candidates also employed cotton rolls. Age, sex, and gender of the patient have an essential effect on the shade selection as well, as 87% of participants in our study agreed. A study by Alruwaili et al. corroborated this finding that gender and age affected shade selection with a proportion of 74.8%. Ramesh S. and Kumar IL both had 46.2%, with sunlight having the highest percentage at 44.7%. In our survey, 85.60% of participants thought choosing the shade at the beginning was preferable, and more than half (57%) of them favored matching the shades before getting started. Kumar IL and Ramesh S agreed. Regarding asking the patient's viewpoint during shade matching, 84.2% of respondents indicated that they did so, and 79% of respondents in the Kumar IL and Ramesh S studies agreed. In our poll, 41.9% of respondents selected the color "A2."

Our study's findings indicate that "aesthetics correction" is the most frequent concern patients have regarding anterior teeth restoration. Patients are currently asking for aesthetic replacements that must match their current dentition, as Kumar IL and Ramesh S discovered. One of the most important steps in cosmetic restorative dentistry is choosing an accurate shade match, but doing so has always been difficult. As comprehensive color training has remained absent from the dental school curriculum, dentists have little to no training in vision physiology or color science. Dental schools [15-16] do not adequately prepare dental students for teaching people of color. For better and more satisfying shade-matching outcomes in this era of growing interest in cosmetic dentistry, there is a need for adequate training and communication.

The study limitations include that limited sample size was taken, and a longitudinal study should have been planned. The determination of shade and its knowledge is a part of the expertise, and hence a lecture should have been arranged before the survey. Also, questionnaire-based studies generally are unable to paint accurate pictures with respect to a domain of study such as cheiloscopy. Moreover, we would also like to mention the fact that the sample size for our investigation is somewhat limited in comparison to other studies (even related to different fields of research) as far as questionnaire-based studies are concerned. Therefore, more investigations like these, on the same objectives as ours with an increased sample size in consideration need to be performed to ascertain and validate the findings obtained through our study.

## Conclusions

Tooth shade matching has necessary components of subjective and objective standards. The clinical importance of a proper shade selection decision as a valuable adjunct in aesthetic dentistry cannot be overlooked until an appropriate tooth shade is selected for restoration's final outcome.

This investigation draws attention to the fact that dentists receive little to no formal instruction in eye physiology or color science as part of their dental school curriculum. For better and more gratifying shade-matching outcomes in this period of increased interest in cosmetic dentistry, there is a need for sufficient training and communication. The authors would like to conclude that as the color and look of teeth are complex phenomena influenced by numerous factors, greater attention should be paid to enhancing knowledge of color science and its application in aesthetic dentistry. Therefore, the dentist should take into account all potential factors that could affect shade selection to get a satisfactory aesthetic result.
